# Do we need standardized management after termination-of-resuscitation attempts? Autoresuscitation in a 67-year-old woman

**DOI:** 10.1186/s13049-023-01137-2

**Published:** 2023-10-26

**Authors:** Janina Pasierski, Gian-Reto Kleger, Paul Imboden

**Affiliations:** 1 Department of Internal Medicine, Cantonal Hospital St. Gallen, 9007 St. Gallen, Switzerland; 2 Department of Anesthesiology and Intensive Care Medicine, Cantonal Hospital St. Gallen, St. Gallen, Switzerland; 3 Department for Intensive Care Medicine, Cantonal Hospital St. Gallen, St. Gallen, Switzerland; 4 Paramedic Emergency Department, Emergency Physician (SGNOR), St. Gallen, Switzerland

**Keywords:** Autoresuscitation, Lazarus phenomenon, Cardiopulmonary resuscitation, Emergency medicine, Termination of resuscitation

## Abstract

**Background:**

Autoresuscitation is the phenomenon of spontaneous return of circulation after cessation of CPR, also known as the Lazarus phenomenon. Most of the evidence is based on case reports and a few systematic reviews. The occurrence of autoresuscitation may lead to self-reproach and dismay in affected emergency personnel and may rise questions about the correct procedure after terminating resuscitative efforts. In contrast to existing cardiac arrest guidelines there is no standardized approach to terminating resuscitative attempts.

**Case:**

We report a case of out of hospital autoresuscitation in a 67-year-old female after 60 min of advanced cardiac life support. After shock refractory shockable rhythm, we recorded pulseless electrical activity and fixed pupils, consequently resuscitation was terminated. About 50 min later the patient surprisingly showed signs of life. Due to the suggestive history a coronary angiography was performed, showing severe coronary heart disease which necessitated surgical intervention. After ACBP surgery and intensive care followed by treatment on the cardiological ward, she was finally discharged to neurological rehabilitation.

**Conclusion:**

As already proposed by existing literature, there should be at least a 10-min interval of close monitoring after abandoning CPR. Transport of a deceased patient should only take place after secure signs of death can be detected. Further investigation is needed to determine which patients are most likely to benefit from an extended observation period. Our case reports highlights the difficulties in death declaration and the importance of close monitoring after abandoning CPR.

## Background

Autoresuscitation or the Lazarus phenomenon sounds like a medical fairytale, describing the return of spontaneous circulation after cessation of cardiopulmonary resuscitation efforts. Actually, it was first described in 1982 and since then multiple case reports and literature reviews followed [1]. Not only in adults but also in children cases of autoresuscitation have been reported [2]. Of notice, until now no studies reported the occurrence of autoresuscitation without attempts of life support, although there have been reports of transient resumption of circulation after withdrawal of life-sustaining therapy [3–5]. Most of the documented cases occurred after resuscitation following non-traumatic cardiac arrest [6].

Different mechanisms have been discussed as possible explanations for the unexpected return of circulation. One of the most popular hypothesis postulates that air trapping in the lungs, caused by hyperinflation and excessive tidal volumes, leads to high intrathoracic pressure, thus delaying CPR drugs from reaching the heart [6, 7]. In previous reports autoresuscitation occurred in the presence of variable cardiac rhythms, not only asystole [6, 8, 9].

The latest revised guideline from 2021 for adult advanced life support (ALS) from the European Resuscitation Council (ERC) also briefly mentions autoresuscitation and recommends a ´no touch` period to rule out its possibility without defining a clear time period [10]. Criteria for terminating resuscitation attempts are being discussed in the ´Ethics and end of life decision` part of the latest ERC guidelines. Here, the colleagues refer to the 2020 Liaison Committee on Resuscitation (ILCOR) Consensus on Science and Treatment Recommendation supporting the termination of resuscitation (ToR) rules, also mentioning in brackets, that there is very low certainty evidence for those rules. Those ToR rules include the initial presence of asystole, unwitnessed cardiac arrest, patient age 81 years, unknown no or low-flow time with no lay resuscitation until arrival of emergency personnel as well as absence of ROSC after 14 min of ALS. The ILCOR also recommends that none of these rules should be used solely to determine ToR. The difference between in-hospital and out-of-hospital setting with often very limited access to information sources in the latter is also being discussed. Reduced hospital resources and a reduced number of patients transported to the hospital after cardiac arrest is considered a positive result by the implementation of these rules. [11]

When encountering a lifeless person there is a clear strategy how to manage the situation—but do we have a precise guideline how to proceed when resuscitation efforts are terminated?

This case report highlights the necessity to establish a guideline not only for the termination of resuscitation attempts (TRA) but also for the procedures after TRA-decision. Here, we would like to propose a strategy on how to proceed after TRA for emergency medical personnel.

## Case report

A 67-year-old woman with no past medical history of heart disease collapsed after expressing chest discomfort while she was taking a walk with a group of friends. Lay resuscitation was immediately started.

After 15 and 20 min respectively the paramedics and emergency physician arrived at the scene and proceeded with advanced cardiopulmonary resuscitation (CPR) according to the current guidelines with a compression-ventilation ratio of 30:2. The initial electrocardiogram (ECG) rhythm was ventricular fibrillation (VF), thus 1 defibrillation at 120 Joule (J) biphasic followed by 5 shocks at 200 J were delivered. Following the fifth shock, the team decided to reposition defibrillator pads from sternal-apical to anterior–posterior. Nevertheless, the rhythm deteriorated into pulseless electrical activity (PEA). An endotracheal tube (ID 7.00 mm) was inserted 25 min after the CPR start using video laryngoscopy. From there, we continued thorax compressions and manual ventilation aiming a tidal volume of 400–500 ml and 10 breaths/minute. No (naso-)gastric tube was used during bag mask ventilation or after endotracheal intubation. A total of 6 mg epinephrine and 450 mg amiodarone were administered according to guidelines.

Capnography was used after endotracheal intubation showing p_et_CO2 between 20 and 25 mmHg (= 2.6–3.3 kPa). CPR was continued manually because of the manpower on site, despite the availability of an automated CPR device (AutoPulse®). The pulse checks during rhythm analyses were conducted manually. After 60 min of CPR and deterioration of the shockable rhythm into PEA with broad QRS-complexes the patient showed fixed and dilated pupils, the p_et_CO2 remained stationary. Regarding the prolonged CPR and deterioration of a shockable into a non-shockable rhythm the emergency team reached the joint decision to TRA.

The patient remained on the ECG monitor for the next 5 min, the ventilation bag was disconnected from the endotracheal tube, whereas the tube itself remained in situ. Agonal breathing resumed. Since the patient already was in the ambulance at the time the cardiopulmonary resuscitation was abandoned, it was decided that the body should be taken to the hospital for further legal medical inspection. All monitoring devices were removed.

About an hour after TRA, the patient surprisingly showed signs of life, spontaneously moved her head, but did not respond to voice or pain. A subtle radial pulse was palpable, she had a systolic blood pressure of 60 mmHg, peripheral oxygen saturation of 70% and a normal heart rate. The patient immediately received catecholamines to stabilize circulation, ventilation was restarted with FiO2 1.0 and sedation and muscle relaxation were administered. About 5 min after clinical recognition of autoresuscitation and stabilization of vital signs the patient arrived at the emergency department. Due to the suggestive history a coronary angiography was performed, showing a marked three-vessel coronary heart disease with a subtotal stenosis of the principal trunk as well as a high-grade stenosis of the middle segment of the left anterior descending artery (LAD). She was planned to receive aortocoronary bypass (ACB). Before scheduling for ACB, sedation was reduced to investigate the extent of neurological damage. When she showed horizontal gaze and spontaneously moved her limbs, this was interpreted as a sign of potentially good outcome.
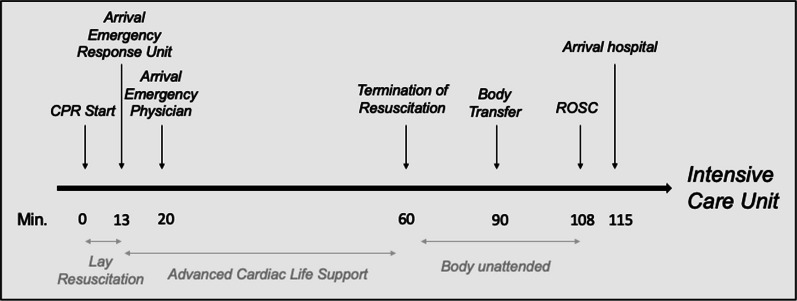


After the surgical intervention she developed acute right heart failure, which was treated by intra-aortic balloon pump and open chest. A week later she was successfully extubated. After all sedatives were stopped, adequate neurologic contact could be established while pronounced weakness due to critical illness polyneuropathy was present.

An initially existing unsteady gait as well as cognitive impairment in the sense of intermittent confusion and mental slowdown showed itself decreasing under constant physical and ergo-therapy. At day 19 after autoresuscitation, she was finally discharged to a neurological rehabilitation unit. After another month the patient left the medical facility as a self-employed pedestrian showing age-appropriate cognitive performance.

## Discussion

We report a case of prolonged CPR, TRA and delayed ROSC in the sense of autoresuscitation. The elapsed time between TRA and the recognition of ROSC was almost an hour. So far, this is the longest reported period of autoresuscitation with a positive patient outcome [6]. In contrast to our case report, a new review article by Zorko et al. showed that autoresuscitation was reported to occur between one and 20 min after circulatory arrest [12]. However, the timing of ROSC in our case remains unclear as the patient lacked continued monitoring.

The underlying medical reasons for the phenomenon of autoresuscitation are speculative and are based on case reports and a few systematic reviews. Hyperinflation of the lungs is a possible culprit that can lead to high intrathoracic pressure, caval and right heart compression and may hinder venous return to the heart [6, 13]. In our case, we did not use a mobile ventilator but delivered manual ventilations which could have led to higher tidal volumes and a higher ventilation rate than the proposed 10/Minute. It’s possible, that an associated auto-PEEP led to a delayed venous return, preventing the CPR drugs given per protocol to reach the site of action. The insertion of a gastric tube could have had a decompressing effect on the potentially high intrathoracic pressure but was not used in this case.

After abandoning further CPR, a final central pulse check was done by an emergency rescuer and the physician. ECG and capnography were removed within 5 min after TOR while leaving the endotracheal tube in situ. The tube could have worked as an airway-splinting and thus facilitated breathing.

We also mentioned the availability of an automated compression device (AutoPulse®), which was not used because of the manpower on site. Because of the shock refractory shockable rhythm transport to the cardiac center during CPR could have been a rational alternative. Shortly after telephone consultation with the anaesthesiologist on duty the decision was made to stay on site because of the change from shockable to non-shockable rhythm and the length of CPR. Our local regulations allow a very restrictive use of this automated CPR device due to skepticism regarding application security. In 2017 Koster et al. discussed the safety for mechanical chest compression devices and described a non-inferiority for the LUCAS® device whereas for the AutoPulse® severe or life-threatening damage could not be excluded. They also stated that there is an association between CPR duration and more bone and visceral damage. In our patient, only a fracture of the fifth rib on the right was revealed by CT scan. Finally, good quality manual chest compressions are not inferior to the use of automated CPR devices. [14, 15]

As mentioned in the case report section of the manuscript, the pulse checks were carried out manually due to the lack of a prehospital ultrasound device. It’s possible that the use of Point-of-Care Chest Ultrasound (PoCUS) could have shown remaining minimal cardiac activity and thus prompted ongoing clinical evaluation and continuation of monitoring. PoCUS may probably improve the recognition of persisting cardiac activity and is easy to learn [16]. On the other hand, ultrasound devices are not universally available, will need trained emergency service personnel and are not universally diagnostic because of anatomic reasons. But as PoCUS can be used for the detection of a variety of pathologies, it should be introduced in the standard equipment of emergency teams.

On the one hand existing literature recommends a minimum CPR length of 20 min, which in our case was much longer with a total of 60 min. On the other hand, the Swiss Academy of Medical Sciences proposes that after 20 min of ALS the chance of survival with good neurological outcome (CPC 1–2) drops to < 1% [17]. In contrast, our patient was discharged as a self-employed pedestrian with age-appropriate neurological performance despite the extended CPR length and the late clinical detection of ROSC.

In contrast to the extensively discussed ToR rules the ERC mentions autoresuscitation briefly in the context of uncontrolled organ donation after circulatory death and recommends a ´no touch` period to rule out its possibility [10]. Unfortunately, no further information is given about how long this period should be and what measures should be taken in order to not miss the occurrence of autoresuscitation. This reflects the complex and yet unclear situation regarding the correct procedure after TRA.

In emergency medicine we rely on various guidelines from different medical societies. There are recommendations how we should take care of patients who suffer from dyspnea, chest pain or even cardiac arrest. But until now we do not have a standardized approach when it comes to termination of resuscitative efforts. In this precarious situation the emergency personnel have to deal with different requirements all at once. A decision has to be made if there should follow a legal inspection of the body, relatives need to be taken care of or maybe the alert for the next rescue mission is coming in. And somewhere in between all of that we have to take care of the actual patient. Most of our medical tasks are complex, and so is death determination. In the clinical setting, especially before organ donation, two experienced physicians from different specializations are needed to accomplish this task [18].

It’s very likely, that a longer monitoring period and a standardized approach to TRA would have led to an earlier recognition of ROSC, faster treatment by specialists and less distress in affected emergency care givers.

The absence of a central pulse via manual pulse check and cardiac auscultation is an insecure tool to exclude minimal cardiac activity after TRA [19]. Therefore, we propose to monitor the patient via ECG and—if intubated—capnography. Any existing airway device should be disconnected from the ventilator or ventilation bag to enable escaping of trapped air. After at least 10 min the absence of brain stem reflexes should be checked. This includes the absence of light reactions to the pupils, corneal reflexes, reaction to painful stimuli, gag reflex and cough reflex. Additionally, patients with persistent gasping should remain under surveillance as long as gasping persists.

Finally, transport of a deceased patient should be undertaken after detection of indisputable signs of death, such as livores.

## Conclusion

Our medical responsibility does not end after withdrawal of life support.

This case highlights the necessity of a standardized management after TRA. As already proposed by the existing literature, there should be at least a 10-min interval of close surveillance after TRA [6, 7].

Therefore, we propose to use the following checklist:
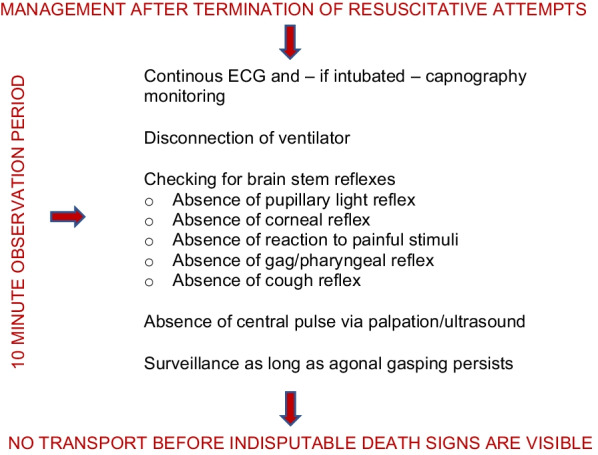


## Data Availability

The data used to support the findings of this case study are included within the article.
